# Photocatalytic efficiency under visible light of a novel Cu–Fe oxide composite films prepared by one-step sparking process

**DOI:** 10.1038/s41598-022-08244-7

**Published:** 2022-03-10

**Authors:** Arisara Panthawan, Nidchamon Jumrus, Panupong Sanmuangmoon, Winai Thongpan, Tewasin Kumpika, Wattikon Sroila, Ekkapong Kantarak, Adisorn Tuantranont, Pisith Singjai, Wiradej Thongsuwan

**Affiliations:** 1 PhD’s Degree Program in Materials, Faculty of Science, and Graduate School in Chiang Mai University (GSCMU), Chiang Mai, Thailand; 2 Center of Excellence in Materials Science and Technology, Chiang Mai University, Chiang Mai, Thailand; 3 Department of Physics and Materials Science, Faculty of Science, Chiang Mai University, Chiang Mai, Thailand; 4 Faculty of Science, Chiang Mai University, Chiang Mai, Thailand; 5 Thailand Organic and Printed Electronics Innovation Center, National Electronics and Computer Technology Center, National Science and Technology Development Agency, Klong Luang, 12120 Pathumthani Thailand; 6 Research Center in Physics and Astronomy, Faculty of Science, Chiang Mai University, 239, Huay Kaew Road, Muang, Chiang Mai, 50200 Thailand

**Keywords:** Energy science and technology, Materials science, Nanoscience and technology

## Abstract

Copper–iron (Cu–Fe) oxide composite films were successfully deposited on quartz substrate by a facile sparking process. The nanoparticles were deposited on the substrate after sparking off the Fe and Cu tips with different ratios and were then annealed at different temperatures. The network particles were observed after annealing the film at 700 °C. Meanwhile, XRD, XPS and SAED patterns of the annealed films at 700 °C consisted of a mixed phase of CuO, γ-Fe_2_O_3_, CuFe_2_O_4_ and CuFe_2_O. The film with the lowest energy band gap (*E*_*g*_) of 2.56 eV was observed after annealing at 700 °C. Interestingly, the optimum ratio and annealing temperature show the photocatalytic activity under visible light higher than 20% and 30% compare with the annealed TiO_2_ at 500 and 700 °C, respectively. This is a novel photocatalyst which can be replaced TiO_2_ for photocatalytic applications in the future.

## Introduction

Photocatalysis is a green technology for environmental purification, in particular the decomposition of organic pollutants^1–3^. Over the last decade, many researchers have been reported that n-type semiconductor materials successfully photodegraded organic pollutants based such as titanium dioxide (TiO_2_)^4–7^. Anywise, n-type semiconductors are still limited due to their large forbidden bands, low quantum yields, and unsuitable conduction band edges^8,9^. Thus, p-type semiconductors have been developed to expand the field of photocatalysis^10^. Copper (Cu) and iron (Fe) oxide are p-type semiconductors that can exhibit much more excellent properties in many applications^10–13^.

Generally, the combinations of metal oxide can produce a novel compound which might improve their physical, chemical, optical and electrical properties, such as Cu–Fe oxides^14–17^. However, the report about Cu–Fe oxides in the field of photocatalysis is infrequently found. In this work, we aim to synthesize novel Cu–Fe oxides composite films by a one-step sparking process. This process has been developed in our lab which can prepare small, uniform particles, high porous films, and determine the composite ratio^18–26^. Moreover, the sparking process requires neither complicated steps nor special equipment, cheap, fast, and non-toxic. Surface morphology, chemical and optical properties of the as-deposited composite films were improved by heat treatment. The effect of heat treatment on morphology, chemical and optical properties were reported and discussed. Furthermore, the photocatalytic activity under visible light between Cu–Fe oxides and TiO_2_ films was examined and compared.

## Experimental details

The experiment was carried out using a high DC voltage of 2.0 kV applied to Fe tips (0.25 mm, purity 99.5%, Advent Research Material Ltd.), and Cu tips (0.25 mm, purity 99.9%, Advent Research Material Ltd, UK). Cu:Fe with the ratios of 4:0, 3:1, 2:2, 1:3, and 0:4 can be defined from the number of sparking heads. The tips were placed 1.5 cm above the quartz substrate (1 × 1 cm^2^) at 1 mm spacing under atmospheric pressure. The nanoparticles were then deposited on the substrate with a deposition rate of 52.33 nm/min for 10 min after sparking off the Fe and Cu tips. The as-deposited films were then annealed at 500, 600, 700, 800, and 900 °C for 60 min to improve their crystallinity.

Morphology, chemical and optical properties of the samples were characterized using scanning electron microscopy (SEM, JEOL JSM300 and SEM, JEOI JSM 6335F), transmission electron microscopy (TEM, JEOL JEM 2010), X-ray Photoelectron Spectroscopy (XPS, AXIS Ultra DLD-X-ray Photoelectron Spectrometer and a monochromatic AlKa X-ray excitation source) and UV–Vis spectroscopy (Hitachi U-4100).

Photocatalytic activity under visible light was investigated by the decomposition of methylene blue (MB) solution (Ajax Finechem). The samples were dipped into 3.0 mL of MB solution with a concentration of 10.0 µM and then irradiated a lamp (TB814SU-Y lamp with wavelength and luminance of 340–900 nm and 6.57 × 10^5^ Lux, respectively) for 1–5 h. Degradation of MB can be indicated by measuring the absorbance using UV–Vis spectrophotometer.

## Results and discussion

**Figure 1:**
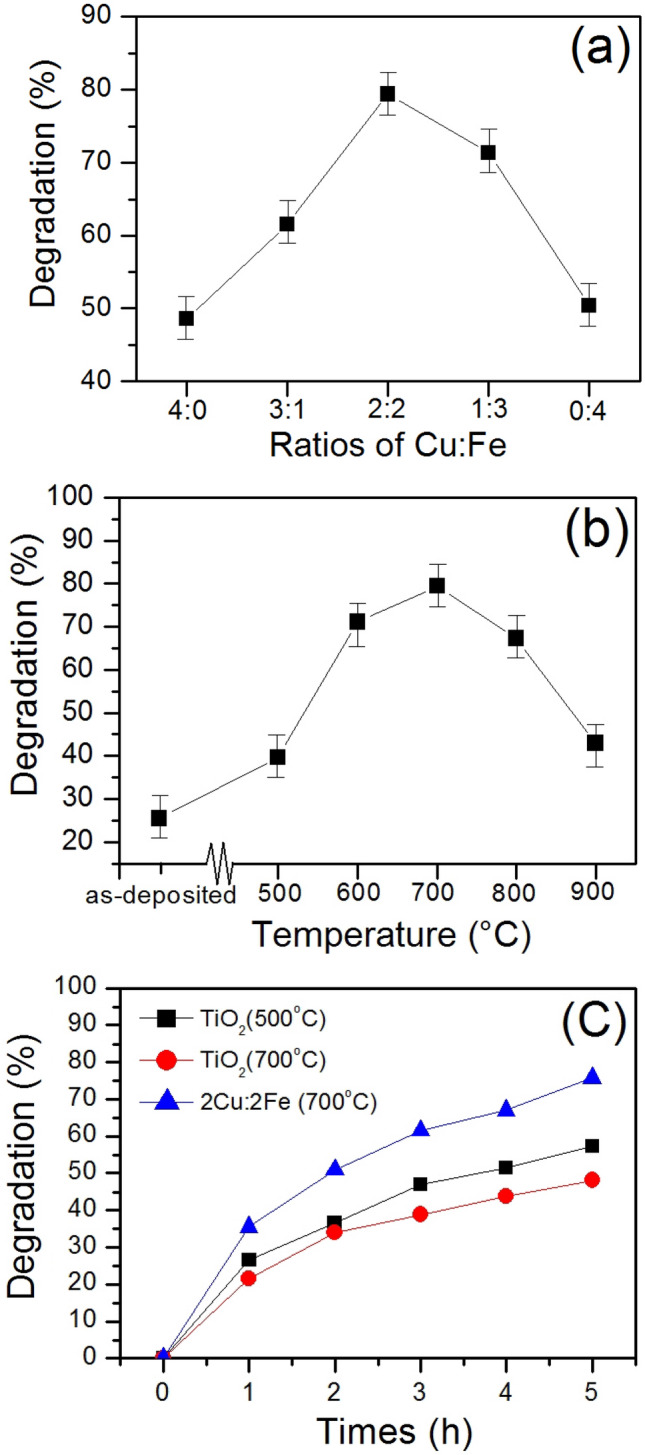
plot of degradation versus (**a**) ratios of Cu:Fe, (**b**) annealing temperatures, (**c**) irradiation time of the Cu–Fe oxide films.

The mass loading of the TiO_2_ and Cu–Fe composite photocatalyst on quartz substrates in this work was 0.12 mg/10 min. This result is in good agreement with our previous papers^26,27^. The effect of Cu:Fe ratio and annealing temperature on MB degradation have shown in Fig. 1a,b. Moreover, the annealed TiO_2_ at 500 °C and 700 °C which were prepared by the sparking process were used to compare MB degradation with the Cu–Fe oxide film at 700 °C against irradiation time, as shown in Fig. 1c. It is noted that the annealed Cu–Fe oxide film at 700 °C with the ratio of 2:2 has a degradation performance higher than 20–30% compared with the well-known photocatalyst such as TiO_2_. Thus, a new finding of Cu–Fe oxide which was used as photocatalyst is a strong point of this work.

**Figure 2:**
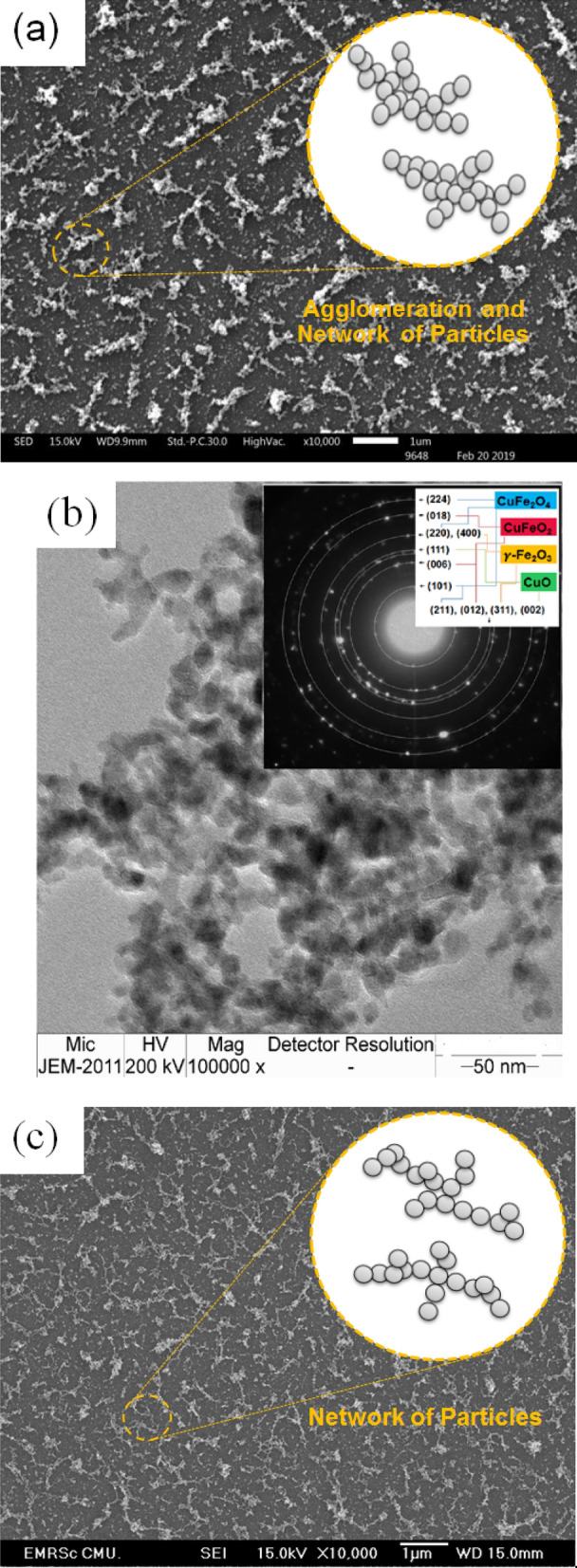
(**a**) SEM image, (**b**) TEM image and their SAED pattern (inset) of the annealed Cu–Fe oxide film at 700 °C with a ratio of 2:2 and (**c**) SEM image of the annealed TiO_2_ film at 500 °C.

Figure 2a shows the morphology of the annealed Cu–Fe oxide film at 700 °C with a ratio of 2:2. The nanoparticles were aligned to networks with the length and width of 1410 nm and 279 nm. This is because of the high surface energy of nanoparticles, Cu–Fe oxide nanoclusters were agglomerated to decrease their surface energy. Meanwhile, the agglomeration of adjoining grains becomes more observable for higher kinetic energy^28,29^. According to the arrangement of the network particles, it can increase the MB decomposition which corresponds to Fig. 1b.

TEM image of the annealed Cu–Fe oxide film at 700 °C with a ratio of 2:2 is shown in Fig. 2b. It is clearly seen that the actual particle sizes are in the range of 4–15 nm, while the energy dispersive x-ray shows the amount of Cu, Fe, and O are 28.26, 31.44, and 40.35 atomic %, respectively (data not shown). Moreover, the selected area electron diffraction (SAED) (inset) shows well-established diffraction rings matching most closely with CuO in the (111), (002) plane (JCPDS 48-0937), $\gamma$-Fe_2_O_3_ in the (311), (220), (400) planes (JCPDS 39-1346), CuFe_2_O_4_ in the (101), (211), (220), (224) planes (JCPDS 34-0425), and CuFeO_4_ in the (006), (012), (018) plane (JCPDS 39-0246). Morphology of the annealed TiO_2_ film at 500 °C is shown in Fig. 2c. The mass loading of ~ 0.12 mg/10 min was obtained on the quartz substrate. The primary nanoparticles were agglomerated to form network particles.

**Figure 3:**
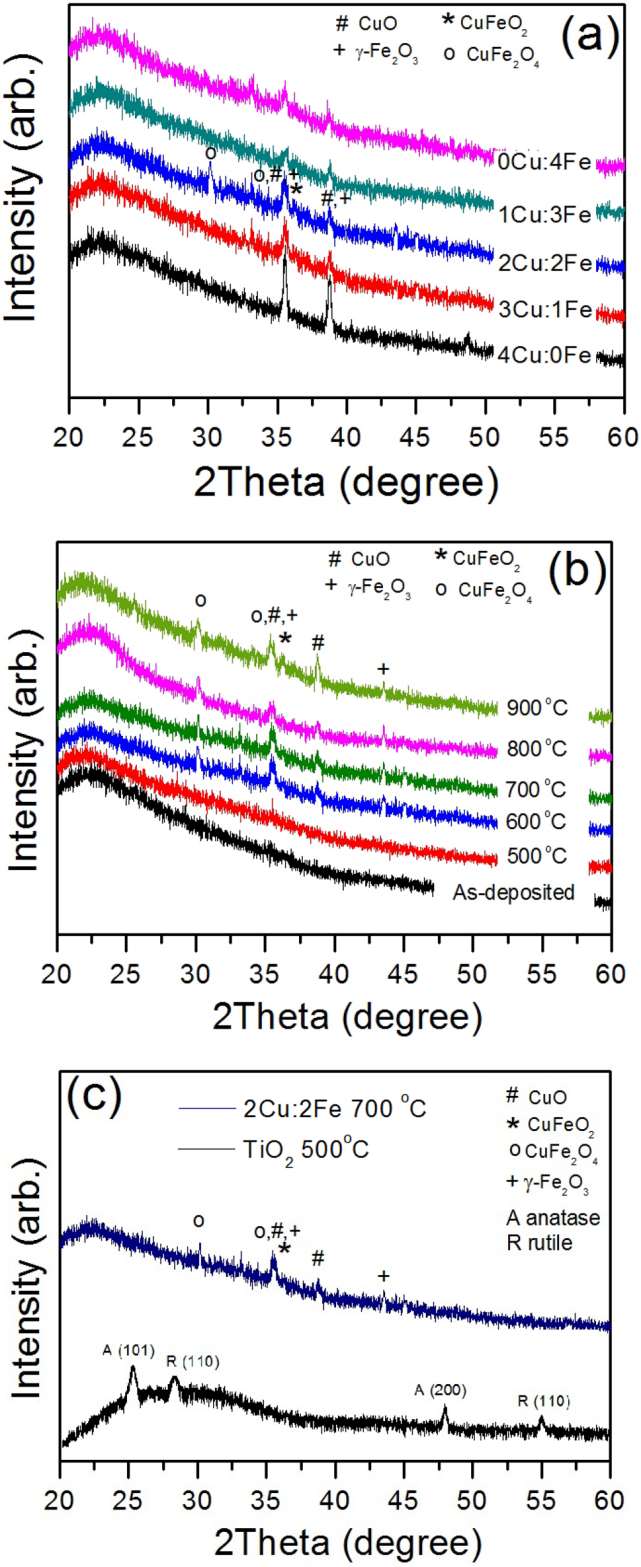
XRD patterns of (**a**) the annealed films at 700 °C with different Cu:Fe ratios, (**b**) the annealed Cu–Fe films with a ratio of 2:2 at different annealing temperatures and (**c**) the Cu–Fe films with optimum ratio and annealing temperature compare with the annealed TiO_2_ film at 500 °C.

Figure 3a shows XRD patterns of the annealed Cu–Fe composite films at 700 °C with various Cu:Fe ratios. It is noted that the ratio has a direct effect on the quantity of each composite film. Interestingly, the ratio of 2:2 shows many Cu–Fe phase compositions which have the highest photocatalytic activity (see Fig. 1a). It might occur the mix-phases can enhance the electronic and optical properties of the films. Figure 3b shows the Cu–Fe composite films with a ratio of 2:2 at different annealing temperatures. No significant peak was observed at the annealed films lower than 600 °C. At 700 °C, the peaks of 30.08° and 35.52° correspond to the (220) and (211) crystal planes of the CuFe_2_O_4_. The peak of 2θ = 35.72° was the (012) crystal planes of the CuFeO_2_. The peaks at 35.56° and 38.77° were the (002) and (111) crystal planes of the CuO. And, the peaks at 35.61° and 43.5° correspond to the (311) and (400) crystal planes of the γ-Fe_2_O_3_. However, the peak at 35° consisted of CuO, γ-Fe_2_O_3_, CuFe_2_O_4_ and CuFe_2_O. While the peaks of anatase and rutile TiO_2_ were observed at 25°, 48° and 27.5°, 55° in the annealed film at 500 °C^22^ (show in Fig. 3c).

**Figure 4:**
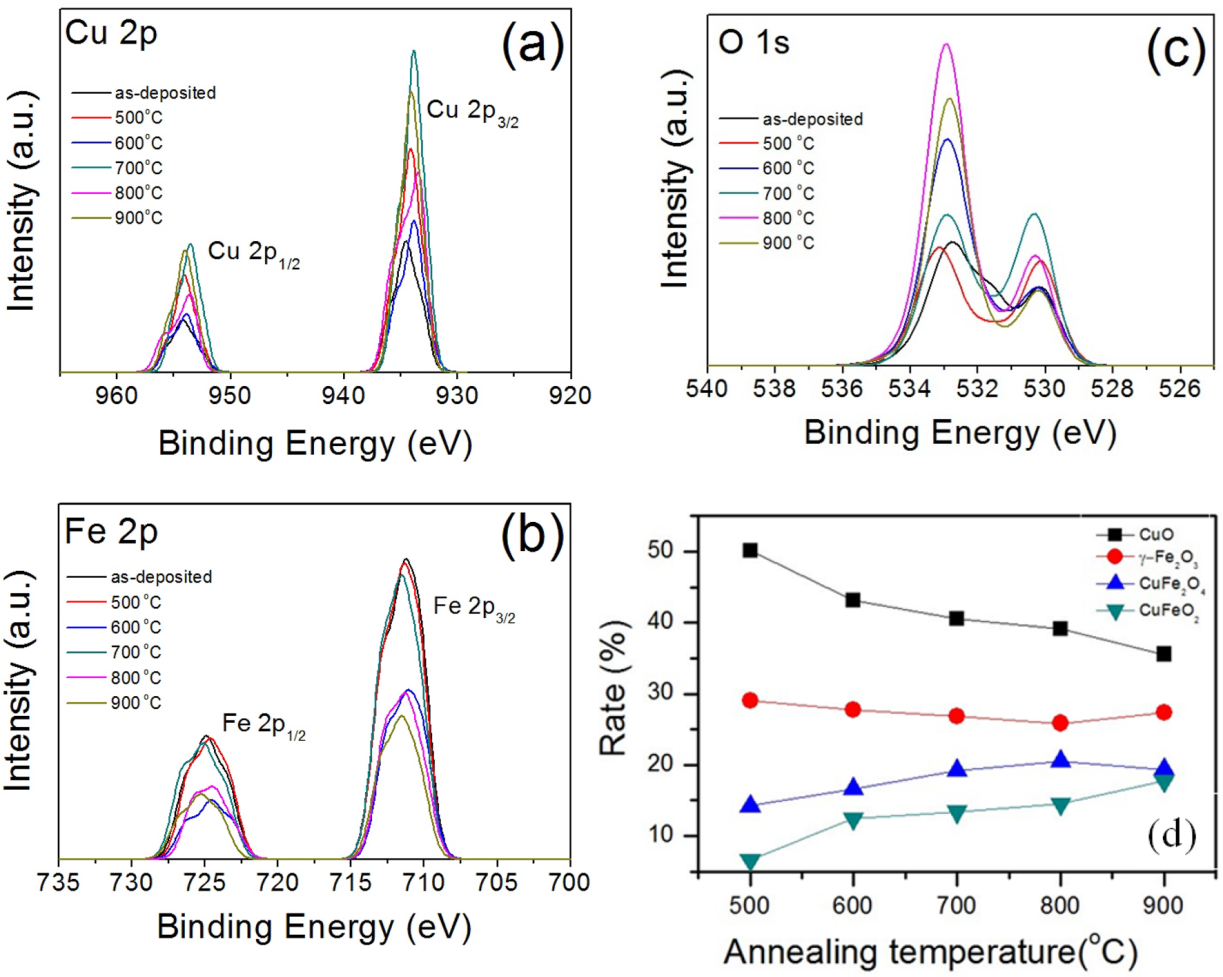
XPS spectra of the Cu–Fe oxide films with different Cu:Fe ratios and annealing temperatures: (**a**) Cu 2p, (**b**) Fe 2p, (c) O 1s, and (**d**) comparison of the Cu–Fe phases at different annealing temperatures.

XPS spectra of the annealed Cu–Fe oxide composite films at 500, 600, 700, 800, and 900 °C for 1 h are shown in Fig. 4. Figure 4a shows symmetric peaks at binding energies of 932.5–934.5 eV and 953.5–954.2 eV assigned to The Cu 2p_3/2_ and Cu 2p_1/2_, respectively. From the figure, the binding energy of 933.7 eV^30^ which corresponds to CuFe_2_O_4_ shows a strong peak after annealing at 700 °C. Whereas the smaller peaks consist of CuO (934.2 eV)^31^ and CuFeO_2_ (932.6 eV)^32^.

The two peaks at 710.5–712.8 eV and 724.5–726.8 eV attributed to Fe 2p_3/2_ and Fe 2p_1/2_ which are characteristic of Fe ions in Cu–Fe oxide films. After the film annealing at 700° C, the highest peak assigned to CuFeO_2_ at 710.6 eV^33^. Moreover, two smaller peaks located at 711.2^34^ and 712.8 eV^35^ corresponds to γ-Fe_2_O_3_ and CuFe_2_O_4_, respectively.

Figure 4c shows the O 1s spectra at various annealing temperatures which can be deconvoluted into three peaks at 532.9^36^, 531.1^37^, and 530.2 eV^38^, which correspond to SiO_2_, Cu(OH)_2_ , and Fe(OH)O, respectively. However, the peak of SiO_2_ (532.9 eV) increased with increasing the annealing temperature. This is due to the agglomeration of network particles at a high temperature can increase the quartz substrate spacing, which corresponds to the SEM result (see Fig. 2a).

The effect of the annealing temperature on the phase transformation can be evaluated by XPS (shown in Fig. 4) are shown in Fig. 4d. It is noted that the CuFe_2_O_4_ and CuFeO_2_ were increased with increasing the annealing temperature^39^. However, an exceed CuFeO_2_ at the annealing temperature higher than 700 °C might inhibit the photocatalytic reactions. This is due to the factors causing the thin films to have an increased energy gap when the temperature is higher than 700 °C because of the effects of % rate of the crystal structure, microstructure characteristics, and the characteristics of chemical compositions^13^.

**Figure 5:**
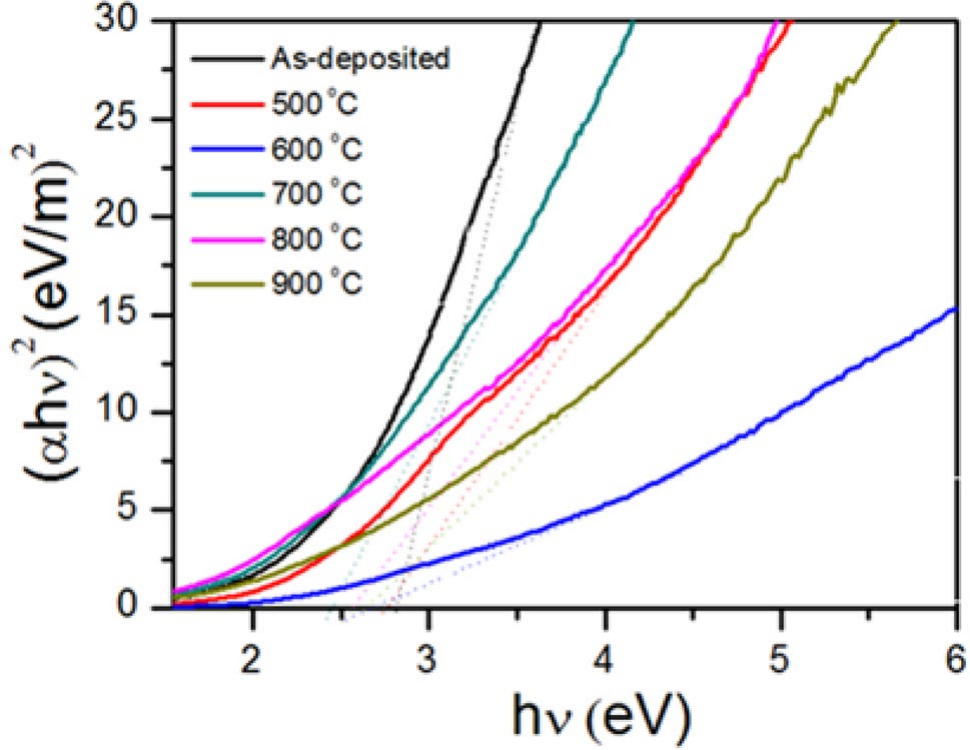
plot of (αhυ)^2^ versus photon energy of the films at different annealing temperatures.

Figure 5 shows the energy band gap (*E*_*g*_) of the as-deposited, the annealed Cu–Fe oxide films at 500, 600, 700, 800 and 900 °C which are 5.35 eV, 3.88 eV, 2.89 eV, 2.56 eV, 2.94 eV and 5.63 eV, respectively. This behavior can be described by an atom distancing increased with the increasing of annealing temperature^40^. Interestingly, the annealing at 700 °C not only shows the lowest *E*_*g*_ but also shows the highest photocatalytic activity (see Fig. 2b). This is because of the good mixing ratio between Cu and Fe oxide phases^41^.

**Figure 6:**
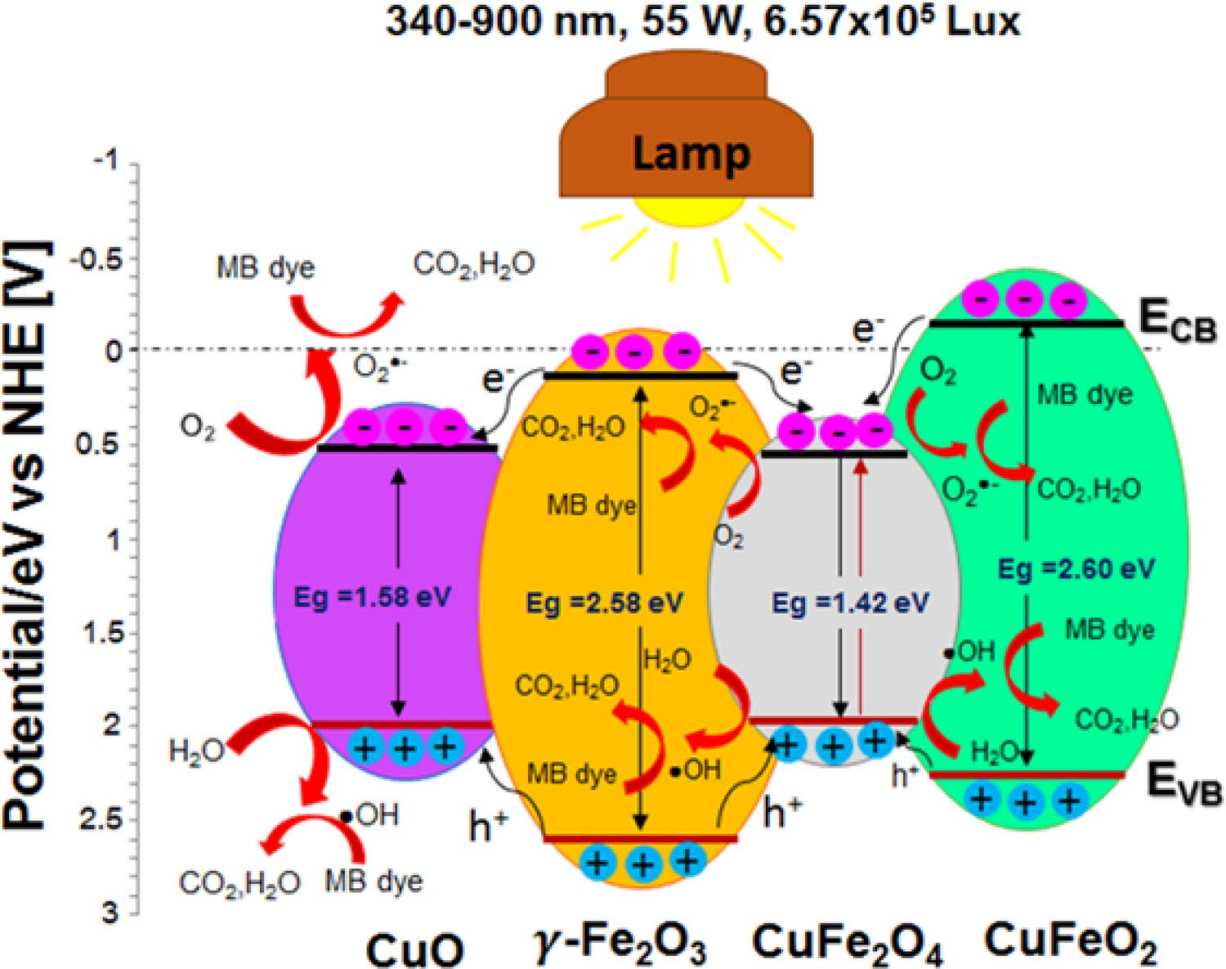
schematic diagram for photocatalytic mechanism of the optimum condition.

Increasing of photocatalytic activity in the annealed Cu–Fe oxide film at 700 °C with a ratio of 2:2 can be described by Cu–Fe mixed-phase mechanism, as shown in Fig. 6. The generation of photocatalytic mechanism is based on pairs of electrons (e^−^) and holes (h^+^) over the composites^42^. The *E*_*VB*_ of CuO, $\gamma$-Fe_2_O_3_, CuFe_2_O_4_, and CuFeO_2_ are + 2.10, + 2.67, + 2.06, and + 2.46 eV/NHE. While, the *E*_*CB*_ of CuO, $\gamma$-Fe_2_O_3_, CuFe_2_O_4_, and CuFeO_2_ are + 0.52, + 0.09, + 0.64 and -0.14 eV/NHE, it can be theoretically calculated using the empirical formula^43^. The possible photocatalytic mechanism of the optimum condition was started by photo-generated electrons (e^−^) and holes (h^+^) pairs from CuFe_2_O_4_ and CuO. The photo-excited e^−^ in the $\gamma$-Fe_2_O_3_ and CuFeO_2_ were injected into the CB of the CuFe_2_O_4_ and CuO. While, the photo-excited h^+^ would transfer to the surface of $\gamma$-Fe_2_O_3_ and CuFeO_2_, which can improve the charge separation and inhibit the e^−^/h^+^ recombination. This is a key factor for the enhancing photocatalytic activity of the annealed Cu–Fe oxide film at 700 °C with a ratio of 2:2 which is greater than a photocatalyst as TiO_2_.

## Conclusions

A novel photocatalyst Cu–Fe oxide films were successfully prepared by a one-step sparking process. The Cu:Fe ratio and annealing temperature are play important role in the photocatalytic efficiency of the Cu–Fe oxide films. This work concluded that the optimum ratio of Cu:Fe and annealing temperature for MB degradation were 2:2 and 700 °C. Moreover, the optimum condition has the photocatalytic efficiency higher than the annealed TiO_2_ at 500 and 700 °C for 20% and 30%, respectively. The results show the Cu:Fe ratio has a direct effect on photocatalytic activity. Furthermore, the annealing temperature not only affects the surface morphology but also affects the *E*_*g*_ and photocatalytic activity. A new finding of this work is the higher performance photocatalyst than TiO_2_ which can be developed and used for photocatalytic applications in the future.
